# Pulmonary lesion subtypes recognition of COVID-19 from radiomics data with three-dimensional texture characterization in computed tomography images

**DOI:** 10.1186/s12938-021-00961-w

**Published:** 2021-12-05

**Authors:** Wei Li, Yangyong Cao, Kun Yu, Yibo Cai, Feng Huang, Minglei Yang, Weidong Xie

**Affiliations:** 1 Key Laboratory of Intelligent Computing in Medical Image (MIIC), Northeastern University, Ministry of Education, Shenyang, China; 2 School of Computer Science and Engineering, Northeastern University, Shenyang, China; 3 Biomedical and Information Engineering School, Northeastern University, Shenyang, China; 4 Neusoft Medical System Co., Ltd., Shenyang, Liaoning China

**Keywords:** COVID-19, Lesion subtypes, 3D texture feature, Random forest, Hybrid adaptive feature selection, Radiomics

## Abstract

**Background:**

The COVID-19 disease is putting unprecedented pressure on the global healthcare system. The CT (computed tomography) examination as a auxiliary confirmed diagnostic method can help clinicians quickly detect lesions locations of COVID-19 once screening by PCR test. Furthermore, the lesion subtypes classification plays a critical role in the consequent treatment decision. Identifying the subtypes of lesions accurately can help doctors discover changes in lesions in time and better assess the severity of COVID-19.

**Method:**

The most four typical lesion subtypes of COVID-19 are discussed in this paper, which are GGO (ground-glass opacity), cord, solid and subsolid. A computer-aided diagnosis approach of lesion subtype is proposed in this paper. The radiomics data of lesions are segmented from COVID-19 patients CT images with diagnosis and lesions annotations by radiologists. Then the three-dimensional texture descriptors are applied on the volume data of lesions as well as shape and first-order features. The massive feature data are selected by HAFS (hybrid adaptive feature selection) algorithm and a classification model is trained at the same time. The classifier is used to predict lesion subtypes as side decision information for radiologists.

**Results:**

There are 3734 lesions extracted from the dataset with 319 patients collection and then 189 radiomics features are obtained finally. The random forest classifier is trained with data augmentation that the number of different subtypes of lesions is imbalanced in initial dataset. The experimental results show that the accuracy of the four subtypes of lesions is (93.06%, 96.84%, 99.58%, and 94.30%), the recall is (95.52%, 91.58%, 95.80% and 80.75%) and the *f*-score is (93.84%, 92.37%, 95.47%, and 84.42%).

**Conclusion:**

The three-dimensional radiomics features used in this paper can better express the high-level information of COVID-19 lesions in CT slices. HAFS method aggregates the results of multiple feature selection algorithms intersects with traditional methods to filter out redundant features more accurately. After selection, the subtype of COVID-19 lesion can be judged by inputting the features into the RF (random forest) model, which can help clinicians more accurately identify the subtypes of COVID-19 lesions and provide help for further research.

## Introduction

In December 2019, the 2019 Coronavirus disease (COVID-19) began to spread worldwide [1, 2]. According to statistics from Johns Hopkins University: as of June 8, 2021, 173,533,746 people worldwide have been diagnosed with the virus, and the death toll is 3,734,475 [3]. With the rapid growth of patients of the COVID-19, the shortage of clinicians is increasingly severe. Currently, clinicians mainly use RT-PCR (reverse transcription-polymerase chain reaction) technology to detect RNA in sputum or nasopharyngeal swabs to detect COVID-19 pneumonia. But this method has a certain false-negative rate $40\%$ [4]. Therefore, clinicians will also use chest CT images as a additional diagnostic method to improve the accuracy of COVID-19 detection for confirmed diagnosis. Moreover, the imaging pattern can change rapidly in a short period of time within the treatment process [5]. During the early COVID-19 surgery, Peng et al. [6] found that the COVID-19 lesions in CT had different subtypes, including GGO, cord, solid and subsolid. Zhang et al. [7] believe that different COVID-19 lesion subtypes have their own unique features.

However, the existing work mainly focuses on lesion detection of COVID-19 or its severity assessment. Few studies are paid attention to the classification of lesion subtypes, which ignores the important role of lesion subtypes in the diagnosis of COVID-19 disease. The subtypes identification of lesions in a timely manner can enable clinicians to better assess the patient’s condition and prescribe precise medicines in personality. Zhao et al. [8, 9] pointed out the severity and the symptoms of COVID-19 pneumonia are different from common pneumonia, and lesions and characteristics of it are also different from common pneumonia. So the lesions caused by COVID-19 pneumonia are more worthy of further study. At the same time, if the patient lesion type can be determined more accurately, the doctor can more accurately determine the COVID-19 patient’s condition by referring to the lesion type. In addition, training deep convolutional neural networks to diagnose COVID-19 requires massive CT impression data and huge computing resources, otherwise it is easy to cause under-fitting of the model. As we all know, it is difficult to collect so much real CT images of COVID-19 for experimentation in a short period of time, while the machine learning algorithm can ignore the shortcomings of small amount of data and insufficient computing resources. Moreover, the machine learning methods of classification for COVID-19 are mainly based on features extracting from the 2D CT images in the exist papers.

To address above issues, we propose a novel random forest-based on hybrid adaptive feature selection (HAFS-RF) for CT slices. Thus, HAFS-RF first extracts the features of the lesion based on the CT slices, and filters redundant features by HAFS. Finally, the subtype of the lesion is judged according to the retained characteristics. It is worth mentioning that 3D radiomics features we used can make full use of the advanced information of lesions which is not discussed in present work to the best of our knowledge in the state of art. Therefore, HAFS-RF helps to study the computer-aided diagnosis of COVID-19 lesion subtypes recognition, at the same time, the study can reduce the image reading burdens of radiologists in vast data. The contributions in this paper can be discussed by three aspects.

1. The pilot research work on lesions subtypes of COVID-19 is discussed in this paper, which has never been seen in previous studies so far and may greatly assist doctor diagnosis and evaluating severity of COVID-19 patients more effectively.
2. The 3D texture radiomics analysis method is applied on COVID-19 lesions diagnosis which is better to explore more hidden inner characters within the lesions to help experts better understand the pathological features of COVID-19.
3. Extensive experiments on clinical real-world datasets demonstrate the effectiveness of the proposed model of hybrid adaptive feature selection method. Moreover, we show the capability of the proposed model for the high dimension feature data with serious imbalance problem.

The rest of the paper is organized as follows. This paper first briefly summarizes the related works. We next introduce introduce the method with the composition of the dataset, the characteristics of 3D features, and the feature selection strategy in “Materials and methods” section. The experimental results on the prepared database are discussed in "Results" section. We finally conclude this paper and look forward to the future work in “Conclusion” section .

## Related work

The research work of lesion segmentation has achieved good results in the diagnosis of COVID-19 through machine learning or deep learning methods.

In terms of machine learning, Shi et al. [10] use medical imaging features and clinical features as input, and logistic regression as a classifier to distinguish COVID-19. Barstugan et al. [11] have made improvements in 2D feature extraction. GLCM (gray-level co-occurrence matrix), LDP (local directional pattern), GLRLM (gray-level run length matrix), GLSZM (gray-level size zone matrix), and DWT (discrete wavelet transform) were used to obtain the second-order statistical features for classification of COVID-19. Ozkaya et al. [12] also propose a new method that fuses and ranks deep features for early detection in SVM. Elaziz et al. [13] used the new fractional multi-channel exponent moments (FrMEMs) to extract features from chest X-ray images. Then an improved Manta-ray search optimization based on differential evolution is used to select the most important features and a K-nearest neighbor classifier is used to distinguish COVID-19 . Tuncer et al. [14] proposed a feature generation method called Residual Exemplar Local Binary Pattern (ResExLBP) and used a novel Iterative ReliefF (IRF) for feature selection. In their work, the SVM classifier achieved 100.0% classification accuracy by using tenfold cross-validation.

In addition, some scholars have proposed some deep learning methods for the diagnosis of COVID-19. Zhou et al. [15] segment COVID-19 lesions from CT by using the U-Net segmentation network with a spatial and multi-channel attention mechanism to assist in diagnosis COVID-19. Khan et al. [16] proposed a deep convolutional neural network called Coro-Net based on Xception architecture, which can detect COVID-19 infection from chest X-ray images. Afshar et al. [17] pointed out that CNN is easy to lose the spatial information between image instances, so an alternative framework based on the capsule network is proposed, which can handle small datasets. Khalifa et al. [18] fine-tuned deep transfer learning for limited datasets to detect pneumonia chest X-ray based on generative confrontation network. Minaee et al. [19] trained four popular convolutional neural networks, including ResNet18, ResNet50, SqueezeNet, and DenseNet-121, to identify COVID-19 disease in the analyzed chest X-ray images. He et al. [20] propose a synergistic learning framework for automated severity assessment of COVID-19 in 3D CT images, by jointly performing lung lobe segmentation and multi-instance classification. Xu et al. [21] use a 3D deep learning model to segment candidate infection areas from lung CT images, then score these areas, and finally uses noise or Bayes function to calculate the final confidence score to classify patients as COVID-19, Influenza-A viral pneumonia (IAVP), and not infected.

## Results

In this section, the classifier evaluation criteria are illustrated firstly. Then, we present experimental results achieved by different methods on the evaluation dataset. Finally, the comparative experiments are conducted to prove the influence of data augmentation, 3D features and HAFS.

### Experimental evaluation of HAFS with RF model

We evaluate HAFS with RF (HAFS-RF) model on the collected chest CT images dataset. Table 1 shows the quantitative results achieved by different methods.

**Table 1:** Performance of COVID-19 classification achieved by SVM, KNN, LR, GaussianNB, QDA, RF, HAFS-RF ($\alpha =0.5$)

Method	Label	Precision (%)	Recall (%)	Accuracy (%)	*F*-measure (%)
SVM	1	76.34	**99.42**	82.3	86.37
	2	99.48	62.07	92.31%	76.45
	3	**100.0**	57.75	98.04	73.21
	4	**96.84**	58.2	91.75	72.71
KNN	1	88.04	86.32	85.66	87.17
	2	83.09	83.23	93.19	83.16
	3	78.05	86.49	98.17	82.05
	4	65.98	67.96	87.58	66.96
LR	1	83.46	88.4	83.8	85.86
	2	76.93	75.3	89.83	76.11
	3	66.67	52.11	96.58	58.5
	4	55.86	50.27	83.7	52.92
GaussianNB	1	88.57	59.6	72.88	71.25
	2	42.62	62.72	75.52	50.75
	3	14.64	86.62	76.01	25.05
	4	37.82	10.19	79.89	16.05
QDA	1	**95.14**	36.67	63.72	52.94
	2	39.1	**97.27**	66.82	55.78
	3	**100.0**	**99.3**	**99.97**	**99.65**
	4	43.38	48.66	79.07	45.87
DT	1	91.85	92.57	91.49	92.21
	2	91.53	87.21	95.53	89.32
	3	86.75	90.34	98.89	88.51
	4	78.36	79.93	91.79	79.14
RF	1	89.70	93.31	90.42	91.47
	2	84.92	83.59	93.48	84.25
	3	86.11	87.94	98.79	87.02
	4	80.78	72.53	91.30	76.43
HAFS-RF (our)	1	92.21	95.52	**93.06**	**93.84**
	2	93.17	91.58	**96.84**	**92.37**
	3	95.14	95.8	99.58	95.47
	4	88.43	**80.75**	**94.3**	**84.42**

Bold values indicate the maximum value of each type of lesion classification index

From Table 1, we can clearly observe that HAFS-RF achieved an accuracy of (93.06%, 96.84%, 99.58% and 94.3%) for label 1, 2, 3 and 4, respectively, under the condition of $\alpha = 0.5$. Followed by DT (91.49$\%$, 95.53$\%$, 98.89$\%$ and 91.79$\%$), next is RF (90.42$\%$, 93.48$\%$, 98.79$\%$ and 91.30$\%$). The accuracy of the remaining models such as SVM, KNN, LR, GaussianNB, and QDA is much lower than theirs. Obviously, HAFS-RF achieved the best performance. For each method, especially the accuracy of QDA is (63.72$\%$, 66.82$\%$, 99.97$\%$ and 79.07$\%$), the accuracy of label 3 is always the highest, some of them even close to 100. The possible reason for this phenomenon is that although we have enhanced the data in the experiment, the number of the four types of lesions tends to be balanced. However, the number of lesions on label 3 is still the least. On the contrary, the precision value of GaussianNB is (88.57$\%$, 42.62$\%$, 14.64$\%$ and 37.82$\%$), and the fitting ability is seriously insufficient. The reason may be that GaussianNB is prone to under-fitting for a small number of samples.

**Fig. 1:**
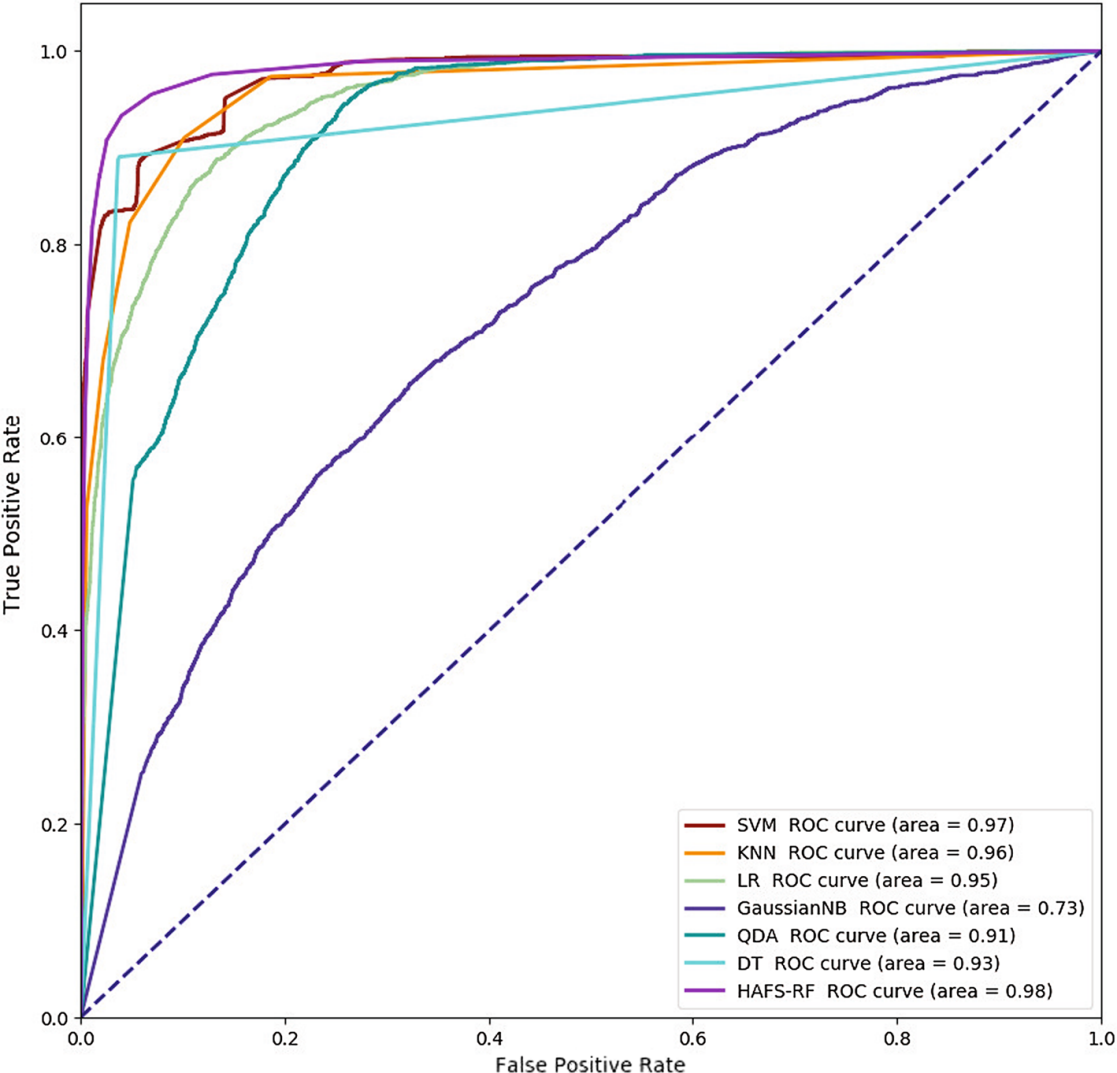
ROC curves achieved by different models

Figure 1 shows the ROC curves of different models. It is also obvious that our method has the highest ROC curve area. These results all show that HAFS-RF can improve the performance and efficiency of COVID-19 classification.

### Influence of data augmentation

To evaluate the effectiveness of the data augmentation, we compare it to without data augmentation, with the results reported in Table 2. The algorithm used for data enhancement is ADASYN, which is a widely used method for the data processing stage before the experiment. The main idea is: firstly, the total number of samples that need to be synthesized are calculated for minority samples. Secondly, for each minority sample, find its neighbors and calculate a weight to automatically determine the number of samples that need to be synthesized for the sample.

**Table 2:** Performance of COVID-19 classification achieved with data augmentation

Augmentation	Label	Number	Precision (%)	Recall (%)	Accuracy (%)	*F*-measure (%)
With	1	2637	93.17	96.85	92.95	94.97
	2	519	89.84	86.02	96.88	87.89
	3	103	89.47	77.27	99.16	82.93
	4	475	82.94	73.25	93.55	77.79
Without	1	2637	92.21	95.52	93.06	93.84
	2	1098	93.17	91.58	96.84	92.37
	3	386	95.14	95.8	99.58	95.47
	4	976	88.43	80.75	94.3	84.42

As can be seen from Table 2, after data augmentation, the number of the four types of lesions changed from (2637, 519, 103 and 475) to (2637, 1098, 386 and 976). The data augmentation consistently achieves better results in label 2, 3 and 4, and worse in label 1. For example, data augmentation achieves (93.84$\%$, 92.37$\%$, 95.47$\%$ and 84.42$\%$) in terms of *F*-measure, none achieve (93.84$\%$, 92.37$\%$, 95.47$\%$ and 84.42$\%$). The possible reason is that the excessive number of samples of label 1 leads to the over-fitting of the model. On the contrary, the insufficient number of other types leads to insufficient fitting ability. After using data augmentation, the four sample sizes are relatively balanced, thus avoiding over-fitting to label 1, so the score of label 1 decreases, but the overall score increases.

### Influence of 3D features

**Fig. 2:**
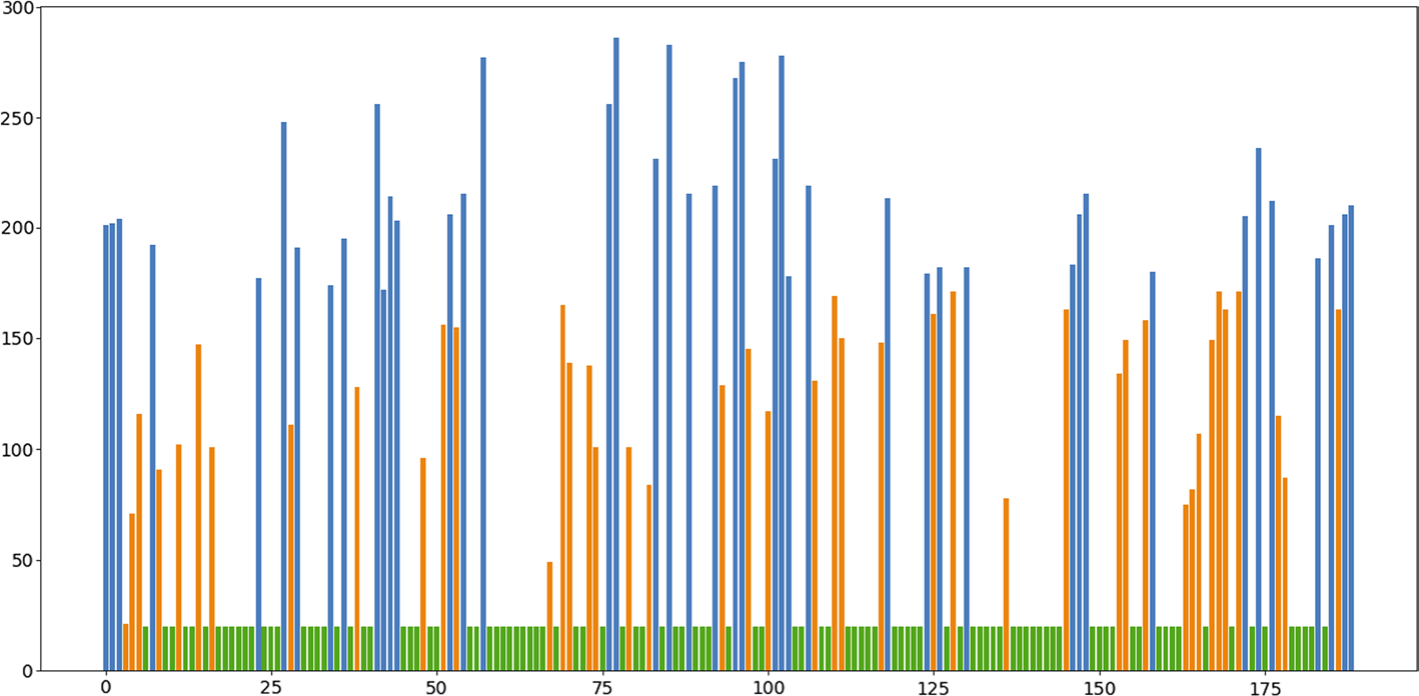
Scores of 189 features

As shown in Fig. 2, each feature will have its own score in HAFS. The green features are discarded in the first stage of HAFS, the orange features are discarded in the second stage of HAFS, and the blue features are selected after HAFS.

The details of blue features of Fig.2 are shown in Table 3. It is obvious that after feature selection, a total of 48 features out of 189 features were retained. Among them, there are 18(6 × 3) 2D features and 30 3D features. More 3D features are retained than 2D features. So 3D features may be more effective than 2D features.

**Table 3:** The features after the process of HAFS

Dimension of features	Kind of features	Characteristics
2D	First order	Length, Mean, Max, Var, ASM, Energy
3D	First order	Robust mean absolute deviation, Mean, Root mean squared, Range, Interquartile range, Skewness
	Glszm	Gray-level variance, High gray-level zone emphasis, Zone percentage, Small area low gray-level emphasis
	Glrlm	Long-run high gray-level emphasis, Difference variance, Gray-level nonuniformity normalized, Run percentage
	Glcm	Sum squares, Id, Joint average
	Gldm	Dependence nonuniformity normalized, Dependence entropy, Dependence entropy
	Shape	Major axis length

To study the influence of 3D features, a comparison was done with and without the 3D features for our model. Results of the evaluated criteria are given for the 189 features in Table 4. As shown in Table 4, HAFS-RF achieves the better classification accuracy when we use 2D and 3D features (93.06$\%$, 96.84$\%$, 99.58$\%$ and 94.3$\%$) than when we use 2D features (89.37$\%$, 93.12$\%$, 98.47$\%$ and 91.2$\%$). The possible reason for improvements is that the 3D features we use have high-level feature representation, thereby improving the typing performance.

**Table 4:** Performance of COVID-19 classification achieved by using 2D features and using 2D and 3D features

Feature	Label	Precision (%)	Recall (%)	Accuracy (%)	*F*-measure (%)
2D	1	88.38	93.56	89.37	90.89
	2	84.18	81.9	93.12	83.02
	3	85.94	79.14	98.47	82.4
	4	79.84	69.29	91.2	74.19
2D and 3D	1	92.21	95.52	93.06	93.84
	2	93.17	91.58	96.84	92.37
	3	95.14	95.8	99.58	95.47
	4	88.43	80.75	94.3	84.42

### Influence of HAFS

To study the effectiveness of the HAFS selection, firstly we compare HAFS with state-of-the-art feature selection methods (*F*-test, MIC, REF, and Lasso). Since they cannot determine the optimal number of features, we select the same number of features of them as HAFS-RF ($\alpha =0.5$) for comparison experiments. The results are reported in Table 5.

**Table 5:** Performance of different feature selection algorithm achieved by *F*-test, MIC, RFE, Lasso, HAFS ($\alpha =0.5$) using Random Forest

Method	Label	Precision (%)	Recall (%)	Accuracy (%)	*F*-measure (%)
*F*-test	1	86.59	91.66	87.32	89.05
	2	78.32	71.99	90.51	75.02
	3	74.62	64.67	97.2	69.29
	4	71.43	67.52	88.66	69.42
MIC	1	87.4	93.09	88.69	90.16
	2	76.75	75.04	90.91	75.89
	3	88.0	70.06	97.98	78.01
	4	73.42	65.59	88.27	69.28
RFE	1	84.82	93.32	86.99	88.87
	2	81.29	77.26	92.28	79.23
	3	88.07	61.15	97.59	72.18
	4	75.43	63.97	88.53	69.23
Lasso	1	87.69	93.44	89.05	90.47
	2	77.84	73.85	91.0	75.79
	3	80.45	68.15	97.52	73.79
	4	74.69	67.69	88.85	71.02
HAFS	1	**92.21**	**95.52**	**93.06**	**93.84**
	2	**93.17**	**91.58**	**96.84**	**92.37**
	3	**95.14**	**95.8**	**99.58**	**95.47**
	4	**88.43**	**80.75**	**94.3**	**84.42**

Bold values indicate the maximum value of each type of lesion classification index

One can observe from Table 5 that compared to the other four methods, HAFS gets the highest accuracy (93.06$\%$, 96.84$\%$, 99.58$\%$ and 94.3$\%$). This proves from the side that the features selected by HAFS are more representative.

Secondly, we further develop four methods based on SVM, KNN, GaussianNB, and QDA by using HAFS (i.e., HAFS-SVM, HAFS-KNN, HAFS-GaussianNB, and HAFS-QDA). We evaluate these eight methods, with the results reported in Table 6.

**Table 6:** Performance of HAFS achieved by SVM, KNN, GaussianNB and QDA by using and not using HAFS

Method	$\alpha$	Label	Precision (%)	Recall (%)	Accuracy (%)	*F*-measure (%)
SVM		1	76.34	99.42	82.3	86.37
		2	99.48	62.07	92.31	76.45
		3	100.0	57.75	98.04	73.21
		4	96.84	58.2	91.75	72.71
HAFS-SVM	0.1	1	90.08	95.03	91.3	92.49
		2	91.64	87.03	95.8	89.28
		3	99.1	77.46	98.92	86.96
		4	84.98	80.14	93.58	82.49
KNN		1	88.04	86.32	85.66	87.17
		2	83.09	83.23	93.19	83.16
		3	78.05	86.49	98.17	82.05
		4	65.98	67.96	87.58	66.96
HAFS-KNN	0.5	1	89.01	87.01	86.6	88.0
		2	83.17	84.52	93.42	83.84
		3	80.77	85.14	98.31	82.89
		4	66.89	69.37	87.97	68.11
GaussianNB		1	88.57	59.6	72.88	71.25
		2	42.62	62.72	75.52	50.75
		3	14.64	86.62	76.01	25.05
		4	37.82	10.19	79.89	16.05
HAFS-GaussianNB	0.1	1	81.77	77.8	77.71	79.74
		2	46.21	51.38	78.19	48.66
		3	34.96	55.63	93.16	42.93
		4	46.67	41.11	80.02	43.71
QDA		1	95.14	36.67	63.72	52.94
		2	39.1	97.27	66.82	55.78
		3	100.0	99.3	99.97	99.65
		4	43.38	48.66	79.07	45.87
HAFS-QDA	0.3	1	86.07	82.19	82.69	84.09
		2	60.85	82.88	84.84	70.17
		3	58.93	92.96	96.68	72.13
		4	56.51	31.84	83.12	40.73

As shown in Table 6, the accuracy of QDA is increased from (63.72$\%$, 66.82$\%$, 99.97$\%$ and 79.07$\%$) to (82.69$\%$, 84.84$\%$, 96.68$\%$ and 83.12$\%$). We can see that HAFS is effective and can improve the performance of the method for different methods. The reason may be that HAFS can select a small number of irrelevant features from a large number of features, thereby avoiding the phenomenon of over-fitting. And we can see that for different models, the best $\alpha$ is different, for example, for SVM, the best $\alpha$ is 0.1, KNN is 0.5, GaussianNB is 0.1, and QDA is 0.3. The possible reason for the difference is that the principle of the classifier is not the same.

## Discussion

For the detection of patients with new coronary pneumonia, the use of machine learning to make judgments is the current mainstream method [10–13]. This is because of the amount of data required for machine learning is small and the characteristics of the lesion can be characterized by extracting its features from the patient’s CT image, the model can be fitted with less data. At present, the subtype classification of COVID-19 lesions, which is similar to the classic classification task, has only been studied by very few people. Therefore, similar to most previous work, in this paper, we also use machine learning methods to classify COVID-19 lesion subtypes.

Compared with the previous method, HAFS-RF has achieved excellent performance. There are two main reasons: (1) for the data set, we use the ADASYN algorithm to enhance the data of different types of samples to alleviate the imbalance of the data in the training set. (2) For each lesion, our group uses more features, including three-dimensional features such as GLCM and LDP, which can better represent the information of the lesion. (3) For the training model, we designed an HAFS feature selection algorithm, which first uses the GA algorithm performs a feature selection, and then uses the *F*-test [32], MINE, RFE [34], and Lasso [35] algorithm to score the features, and selects the head features with higher comprehensive scores, and discards the tail features. Doing so can filter out features with low influence, avoid feature redundancy, and reduce the amount of calculation.

Table 5 shows the performance scores obtained after applying HAFS and other classic feature selection algorithms to our model. It can be seen that the scores obtained by HAFS are the best. It can be seen from Table 1 that the model we used HAFS-RF is better than the traditional RF model, and even scored higher than other models such as SVM.

However, the problem of data imbalance between different subtypes is still difficult to solve, and more CT data will be collected in the next step. In addition, with the increase of COVID-19 image data, deep learning can increasingly exert its superior performance. We will try to use CNN to implement a point-to-point COVID-19 subtype discrimination model in the future. In addition, in our model, the weighting factor $\alpha$ is generally a specific value (i.e., [0, 1.0]). In the future, we will change it to a learnable parameter, so that the performance of the model may be further improved. Finally, it is worth mentioning that the HAFS feature selection method we proposed can be used as a basic method for other machine learning models.

## Conclusion

The most four typical lesion subtypes of COVID-19 are discussed and a computer-aided diagnosis approach of lesion subtype is proposed in this paper. Then the three-dimensional texture descriptors are applied on the volume data of lesions as well as shape and first-order features. The massive feature data are selected by hybrid adaptive selection algorithm and a classification model is trained at the same time. Extensive experiments on clinical real-world datasets demonstrate the effectiveness of the proposed model of HAFS-RF. Moreover, we show the capability of the proposed model for the high dimension feature data with serious imbalance problem. The results show that the 3D radiomics features chosen by HAFS algorithm can better express the advanced information of the lesion data. The classification model obtains a good performance and is compared the models of COVID-19 in the state of art, which can help clinicians more accurately identify the subtypes of COVID-19 lesions and provide help for further research.

## Materials and methods

**Fig. 3:**
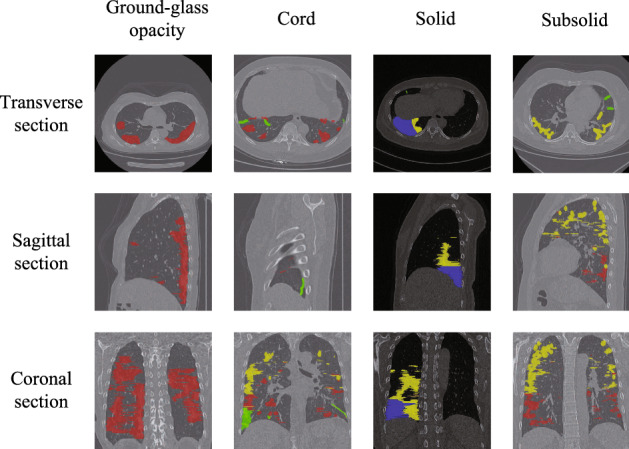
Typical four lesion subtypes in CT images of COVID-19. The labels and regions are given by medical experts. The red area represents ground-glass opacity, the green area represents cord, the blue area represents solid and the yellow area represents subsolid

In this study, four lesion subtypes are studied, namely ground-glass opacity (GGO, referred as label 1), cord (referred as label 2), solid (referred as label 3), and subsolid (referred as label 4) [22]. The CT images of the prepared dataset in this paper are shown in Fig. 3, which are presented in transverse, sagittal and coronal plane, respectively, and the lesions are annotated by two radiologists with ITK-SNAP software.

The numbers of the four types of lesions are 2637, 519, 103 and 475, respectively, and the total is 3734 in original dataset. Table 7 shows the summary of the prepared dataset that are maximum, minimum and standard variance values of the sizes of three directions and volumes for each lesions subtypes. The subtype of ground-glass opacity hosts the majority of COVID-19 which shows imbalance data problem. The subsolid subtype is the largest in size of lesions while the cord subtype is the smallest one.

**Table 7:** Samples of lesion from prepared dataset COVID-19

Label	Num	Statistics	*X*-length	*Y*-length	*Z*-length	Volume
1	2637	Max	247.0	312.0	399.0	20148480.0
		Mean	46.78	49.2	24.84	395826.08
		Std	42.2	50.68	39.63	1596465.23
2	519	Max	186.0	205.0	310.0	5142630.0
		Mean	43.38	41.28	24.86	124041.76
		Std	31.4	27.92	28.17	409901.04
3	103	Max	217.0	301.0	223.0	5878530.0
		Mean	40.46	37.9	21.53	210307.5
		Std	46.06	45.99	26.49	752977.45
4	475	Max	204.0	283.0	378.0	16873920.0
		Mean	61.9	63.26	38.79	722428.14
		Std	50.65	57.84	53.43	1979445.47

**Fig. 4:**
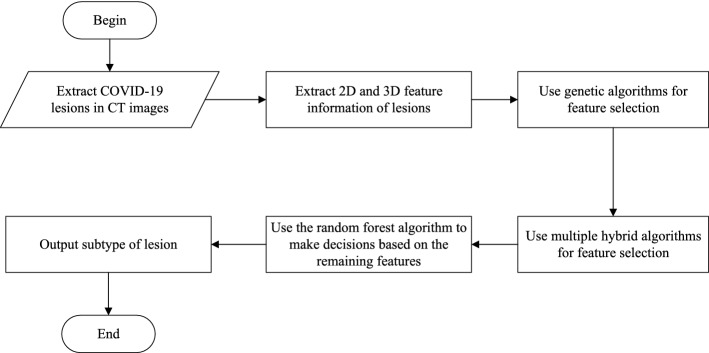
The flowchart of our algorithm

**Fig. 5:**
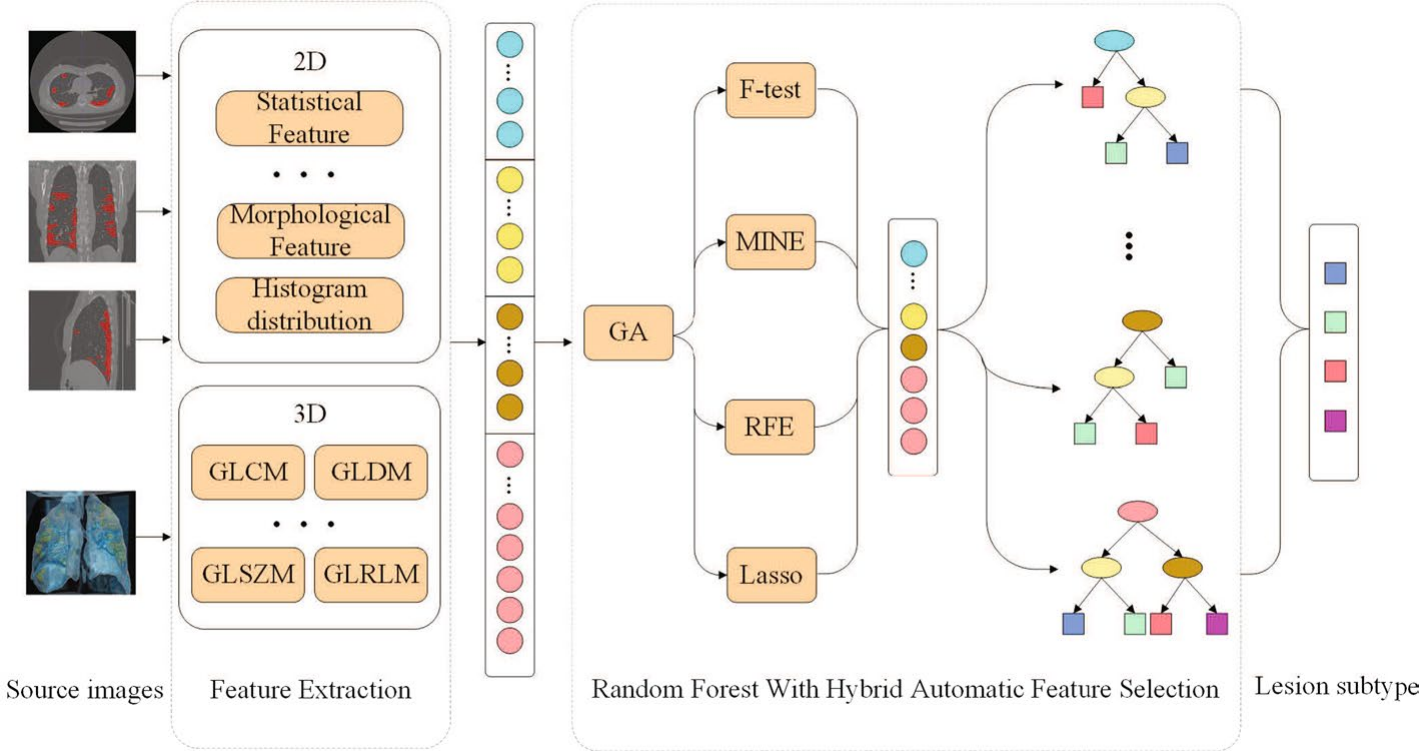
Overview of the COVID-19 classification using random forest based on hybrid adaptive feature selection

Based on this data set, the flowchart of the algorithm is shown in Fig. 4 and the architecture of the algorithm is shown in Fig. 5 which includes four steps. We firstly introduce the algorithm of lesions extraction and data augmentation used in this study. Then, the feature extraction process for the 2D and 3D features are discussed. The implementation details are presented subsequently. Finally, we describe the random forest model in the forth step.

### Lesions data extraction and augmentation

VB-Net is to predict and segment the image of the unknown lesion location. It combines V-Net and bottleneck layers to reduce and combine redundant information [23]. However, all the 3D volume data used in this experiment has the mask position of the corresponding lesion from annotations by the radiologists. Therefore, the VB-Net or V-Net will cause an error in extracting lesions. So we firstly use a breadth-first traversal (BFS) based lesion extraction algorithm to extract lesions which is mostly used in graph structure data. It should be noted that the lesion locations and subtypes are labeled by two radiologists that ensure the accuracy of evaluation data.

The BFS-based lesion extraction algorithm is shown as Algorithm 1. We traverse the mask array, which is created by radiologists, in the entire 3D volume data. When traversing the pixels with a lesion mark, this paper uses the BFS to extract the lesion range from the case with that mask [24]. The input is generally a point in the graph, then use the point to initialize a queue. The main idea of the algorithm is to take out a point from it each time, and then for this point, all nearby points that meet the requirements are enqueued, and then the above process is repeated until the queue is empty. 
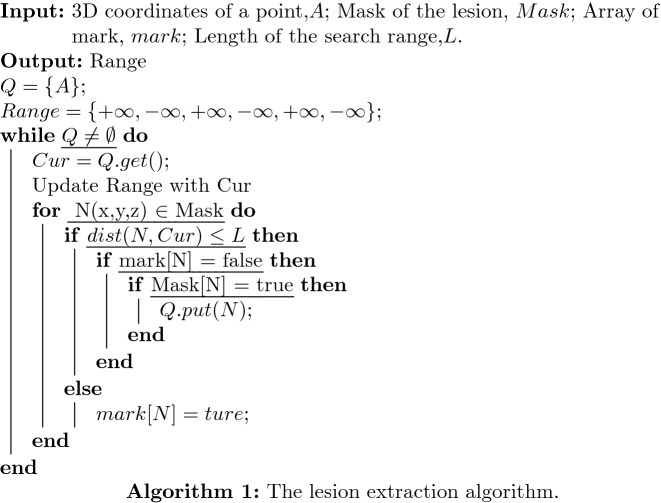


Because the setting of the search range *L* in the algorithm, the algorithm can well avoid the discontinuity of the lesion data in a certain dimension caused by inaccurate data labeling. And we set a global mark in the algorithm, all traversed points will be recorded. There are two advantages to this:

1. In the current BFS process, the marked points will not be enqueued, which can avoid double calculation;
2. In the global traversal of the mask data, the marked points will not call the BFS algorithm repeatedly. This can ensure that each time the BFS algorithm only generates one lesion and returns its 3D ranges.

As we all know, the data imbalance problem will lead to a decrease in the accuracy of multi-classification. The four different subtypes of lesions number are 2637, 519, 103 and 475. Obviously they are unbalanced, and the number of label 1 lesions is far greater than label 3, which can leads to insufficient fitting ability of the classifier to label 3 samples. The unbalance characteristic data can shift the decision boundary of the classifier, and affect the final classification effect. Therefore, we have adopted a data enhancement method based on ADASYN proposed by He [25] to reduce its impact on classification accuracy. This paper adopts a data enhancement method based ADASYN to increase the number of label 2, 3 and 4. The method is briefly introduced in Algorithm 2. 
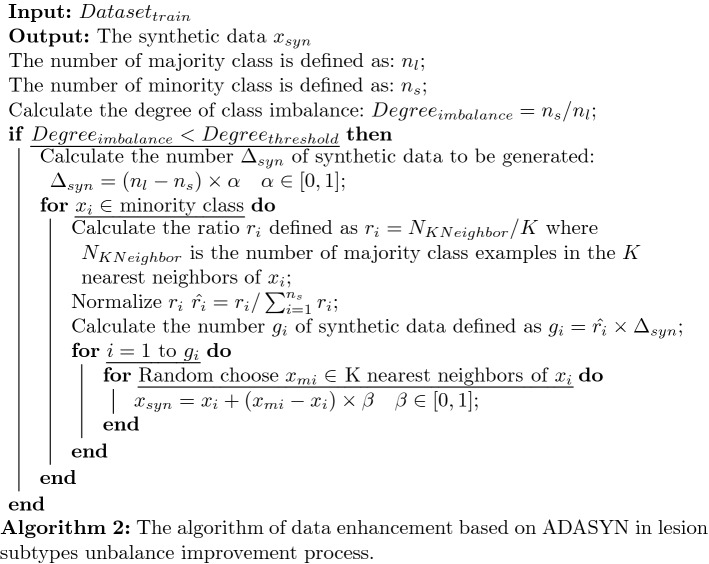


The algorithm increases the number of four subtype lesions from (2637, 519, 103 and 475) to (2637, 1098, 386 and 976) which is evaluated that the data augmentation can effectively improve the classification performance evaluated by the experiments in “Results” section .

### Three-dimensional feature extraction

The most of the current medical imaging research on COVID-19 are mainly based on X-rays images or ignoring the characteristics of CT planar images. Furthermore, 3D features are also seldom considered in the research. Therefore, this paper extracted the 2D features of some certain layers and more 3D features of the CT data to better characterize the lesion information.

The existing methods are mainly based on extracting 2D features from CT images. This ignores that the COVID-19 lesion is a kind of volume data. Therefore, in the process of feature extraction, the connection between layers is ignored, and some hidden features are lost.

In order to improve the accuracy of classification, we extracted multiple types of features of the lesion that include 2D and 3D features and are shown in detail as following:

1. Infected lesions number: The stage of the COVID-19 affects the number of lesions and also affects the distribution of different types. Therefore, we add the total number of lesions in the same patient.
2. Shape features: Some cord-type lesions are significantly different from other lesions in shape, so we extracted three-dimensional shape features from the lesions in order to improve the accuracy of multi-classification.
3. First-order features: The first-order features provide information related to the gray-level distribution of the image. We first obtain the middle layer and the layer with the largest lesion area of CT images in three directions, and extract 14 two-dimensional manual features from them, including mean, var, max, skewness, kurtosis, area, compact, rough, contrast, dissimilarity, homogeneity, energy, correlation, ASM (active shape model). Then we also extract the three-dimensional features and hybrid them into the total features.
4. Second-order features: Second-order features give more information about the relative positions of the various gray levels within the lesion image.
  - The gray-level co-occurrence matrix (GLCM) [26]: The GLCM is a statistical method of analyzing texture that considers the spatial relationship of pixels. The $(i,j){\text{th}}$ element of GLCM $P(i,j|\delta ,\theta )$ describes the number of times the combination of levels *i* and *j* occur in two pixels in the image, that are separated by a distance of $\delta$ pixels along angle $\theta$. The 3D-GLCM considers 13 directions and need to be calculated separately and finally averaged.
  - The gray-level run length matrix (GLRLM) [27]: The GLRLM quantifies gray-level runs, which are defined as the length in number of pixels of the same gray-level value. The $(i,j){\text{th}}$ element of the GLRLM $P(i,j|\delta ,\theta )$ represents the number of runs with gray level *i* and length *j* occur in the image along angle $\theta$. Similar to 3D-GLCM, 3D-GLRLM also needs to be calculated separately for 13 directions.
  - The gray-level size zone matrix (GLSZM) [28]: In the GLSZM *P*(*i*, *j*), the $(i,j){\text{th}}$ element represents the number of zones with gray level *i* and size *j* appear in the image. A zone is defined as the number of connected voxels that share the same gray-level intensity. Contrary to GLCM and GLRLM, the 3D-GLSZM is rotation independent, with only one matrix calculated for all directions.
  - Gray-level dependence matrix (GLDM) [29]: The GLDM quantifies gray-level dependencies in the image. The $(i,j){\text{th}}$ element in GLDM $P(i,j|\alpha )$ equals the number of times a voxel with gray level *i* with *j* dependent voxels in its neighborhood appears in an image. A neighboring voxel with gray level *j* is considered dependent on center voxel with gray level *i* if $|i-j|\le \alpha$. Similar to 3D-GLRLM, the 3D-GLDM is also rotation independent.
  We introduced some 3D features from GLCM, GLSZM, GLRLM, GLDM, and the numbers are 22, 16, 16, 14, respectively. The detailed information of features can be found in the Pyradiomics [30] document on [31]. These features correspond to the GLCM(1–15 16–21 23–24), GLRLM(1–16), GLSZM(1–16), and GLDM(1–14).

In addition, we also extracted 4 features that are width, height, length and volume of the 3D lesion from the bounding box of COVID-19 lesion. In summary, a total of 189 features are used in our study.

### Hybrid adaptive feature selection method

As described in “Three-dimensional feature extraction” section, we extract specific features from 3D lesion data. However, some irrelevant features can easily cause over-fitting of the model, which will reduce the accuracy of the test set. Therefore, before training the model, it is necessary to perform feature selection processing to reduce the influence of the redundant feature.

There are three kinds of algorithms in the existing feature selection process, namely filters, wrappers, and embedded. Although these methods have their advantages, they all have one obvious disadvantage that the number of features in the subset after feature selection cannot be determined. So at this stage, we proposed a Hybrid Adaptive Feature Selection (HAFS) algorithm. It can not only solve the problem of the uncertain number of features in the subset, but also integrate the advantages of various traditional methods.

**Fig. 6:**
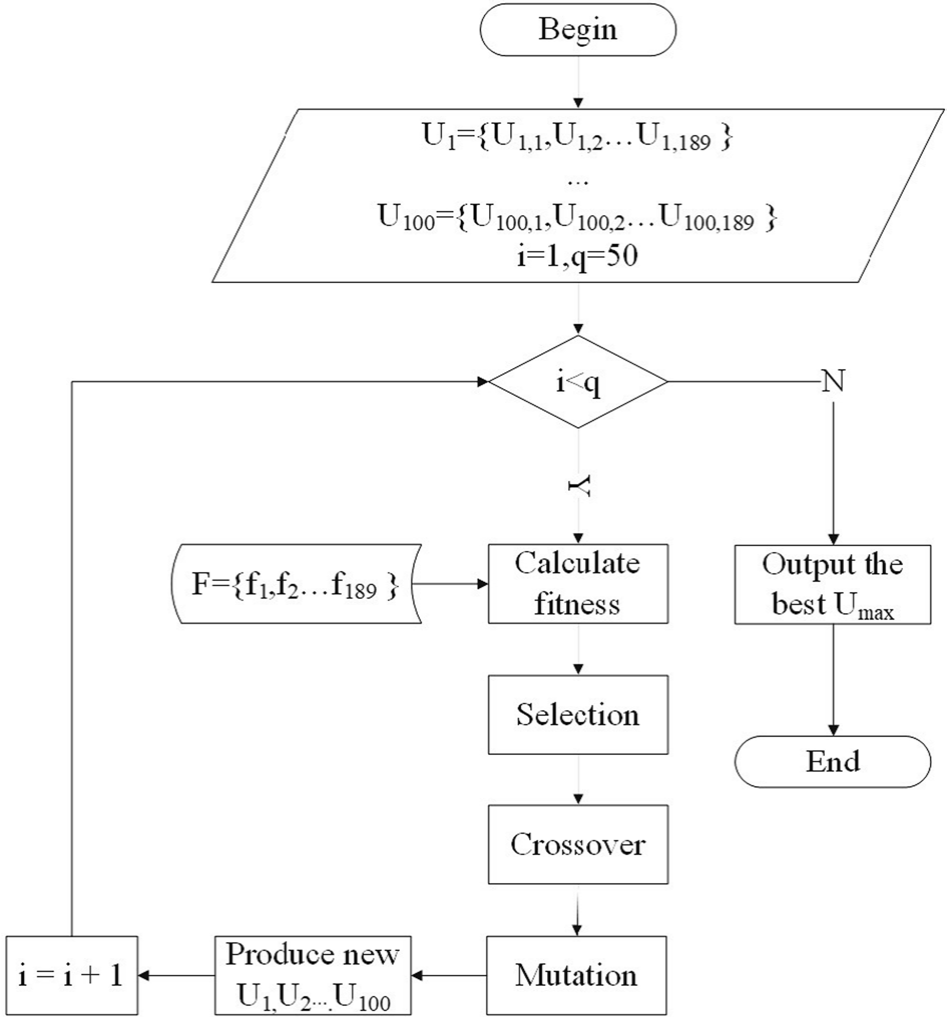
A schematic illustration of first stage of HAFS

HAFS method is divided into two stages. In the first stage, the feature set $F=\{f_1,f_2 \dots f_{189}\}$ is used as input, and the genetic algorithm (GA) is used to accurately select features. First, a population of *q* chromosomes will be initialized, and each chromosome is a binary set of length *n* $U=\{U_1,U_2 \dots U_{189}\}$, each value of $U_i$ is 1 or 0, if $U_i=1$, it means $f_i$ is selected , otherwise, $f_i$ is not selected. Next, *p* iteration will be performed. Before each iteration, individual fitness is evaluated for each chromosome. Then, according to the fitness, the chromosomes in the population are calculated with different probabilities of three genetic operators that are selection, crossover, and mutation. At the end of the iteration, the feature subset $F_{\text{GA}}=\{f_{\text{GA}1}, f_{\text{GA}2} \dots f_{\text{GA}m}\}$ is determined according to the value of each bit according to the chromosome $U_{\text{max}}$ with the highest fitness. A schematic illustration of the first stage is shown in Fig. 6.

In the second stage, in order to integrate the advantages of multiple feature selection methods, we use $F_{\text{GA}}$ as input, and take two filters method that are *F*-test [32] and maximal information coefficient (MIC) [33], one wrappers method that is recursive feature elimination (RFE) [34], and one embedded method that is L1 regularized linear regression model(Lasso) [35] into data preprocessing methods to score features and sort them in ascending order, the results are presented as $F_1, F_2, F_3$ and $F_4$:

1 $${F_1}=  F{\text{-test}}(F_{\text{GA}})=\{f_{F1},f_{F2} \dots {f_{Fm}}\},$$

2 $$\begin{aligned} F_2= \text{MIC}(F_{\text{GA}})=\{f_{M1},f_{M2}\ldots f_{Mm}\}, \end{aligned}$$

3 $$\begin{aligned} F_3=  \text{RFE}(F_{\text{GA}})=\{f_{R1},f_{R2} \dots  f_{Rm}\}, \end{aligned}$$

4 $$\begin{aligned} F_4= \text{Lasso}(F_{\text{GA}})=\{f_{L1},f_{L2} \dots f_{Lm}\}. \end{aligned}$$

So we score each $f_{\text{GA}}$ in $F_{\text{GA}}$ according to $F_1, F_2, F_3$ and $F_4$. According to the $S(f_{\text{GA}})$ score, $F_{\text{GA}}$ is sorted in ascending order. In this way, we can decide which features to keep. The scoring standard, $S(f_{\text{GA}})$, is:

5 $$\begin{aligned} S(f_{\text{GA}})=\frac{\sum _{i=1}^{4}{\text{index}(F_i,f_{\text{GA}})}}{4}. \end{aligned}$$

On the other hand, in order to further prevent feature over-fitting after GA selection, some features of $F_{\text{GA}}$ are selected and a parameter $\alpha$ is set as the ratio of the number of selected features. The final result of feature selection $F_{\text{HAFS}}$ is:

6 $$\begin{aligned} F_{\text{HAFS}} = \alpha F_{\text{GA}}. \end{aligned}$$

### Random forest-based classification model

In the classification stage, the classifier used in this paper is random forest (RF) model. The main idea is: select a subset $S_{\text{b}}$ from all sample set *S* through randomly selecting sample features and sampling. Then a classification and regression tree is established for $S_{\text{b}}$. The classification and regression tree uses the Gini coefficient as the criterion [36]. In this paper, the above process was repeated 1000 times to construct 1000 CART trees to construct a random forest. The random forest-based classification model is shown in Algorithm 3. 
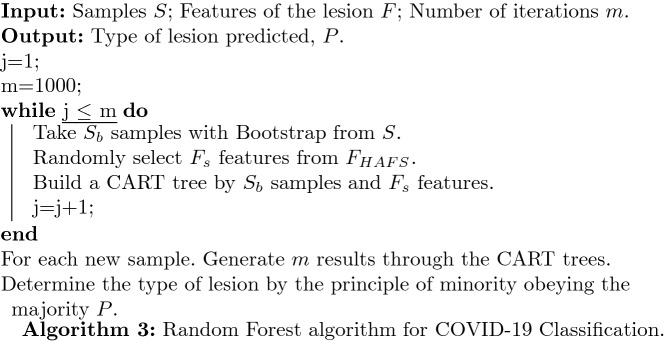


### Experimental settings

Since COVID-19 is a new disease, there are few public data sets of CT images with annotations suitable for this study. Therefore, we extracted lesions from 319 cases of COVID-19 pneumonia patients provided by Neusoft Medical to construct a dataset. All patients received a thin-slice CT scan of the chest by Neusoft 256 slice CT.

The final subsets of features are evaluated by RF classifications associated with tenfold cross-validations. Precision, recall, accuracy and *F*-measure are used to compare the estimated and known labels according to the following expressions:

7 $$\text{Precision}= \frac{\text{TP}}{\text{TP}+\text{FP}},$$

8 $$\text{Recall} = \frac{\text{TP}}{\text{TP}+\text{FN}},$$

9 $$ \text{Accuracy} = \frac{\text{TP} +\text{TN}}{\text{TP}+\text{TN}+\text{FP}+\text{FN}},$$

10 $$\begin{aligned} F\text{-measure} = \frac{2 * \text{Precision} * \text{Recall}}{\text{Precision}+\text{Recall}}, \end{aligned}$$

where TP, TN, FP and FN in Eqs. 7–10 represent true positive, true negative, false positive, and false negative, respectively.

In our experiments, we compare our model with the following widely adopted machine learning methods. These models are relatively classic and commonly used models in the field of machine learning.

1. Logistic regression (LR)
2. Support vector machine (SVM) (use a radial basis function kernel with default parameters.)
3. K-neighbors classifier (KNN) (set $k = 20$ through cross-validation)
4. Decision tree classifier (DT)
5. GaussianNB
6. Quadratic discriminant analysis (QDA)
7. Random forest (RF)

## Data Availability

The patient population data used to support the findings of this study have not been made available because the data are supplied by Neusoft under license and so cannot be made freely available. Requests for access to these data should be made to the corresponding author.

## Abbreviations

CT: Computed tomography
RT-PCR: Reverse transcription-polymerase chain reaction
GGO: Ground-glass opacity
GLCM: Gray-level co-occurrence matrix
LDP: Local directional pattern
GLRLM: Gray-level run length matrix
GLSZM: Gray-level size zone matrix
IRF: Iterative ReliefF
IAVP: Influenza-A viral pneumonia
BFS: Breadth-first traversal
GLDM: Gray-level dependence matrix
GA: Genetic algorithm
MIC: Maximal information coefficient
RFE: Recursive feature elimination
RF: Random forest
KNN: K-Neighbors classifier
DT: Decision tree
QDA: Quadratic discriminant analysis
